# Geographical and climatic contributions to melioidosis hotspot formation in Southern Taiwan

**DOI:** 10.1371/journal.pntd.0012958

**Published:** 2025-04-10

**Authors:** Kuang-Yueh Chen, Kuang-Ying Chen, Hsin-Ping Ho, Hsi-Hsun Lin, Bing-Mu Hsu, Yao-Shen Chen, Duen-Wei Hsu, Chung-Yuan Ren

**Affiliations:** 1 Department of Physics, National Kaohsiung Normal University, Kaohsiung, Taiwan; 2 Department of Biomedical Sciences, National Chung Cheng University, Chiayi, Taiwan; 3 College of Medicine, National Yang Ming Chiao Tung University, Taipei, Taiwan; 4 Department of Biotechnology, National Kaohsiung Normal University, Kaohsiung, Taiwan; 5 Division of Infectious Diseases, E-Da Dachang Hospital, I-Shou University, Kaohsiung, Taiwan; 6 Institute of Clinical Medicine, National Yang Ming Chiao Tung University, Taipei, Taiwan; 7 Department of Earth and Environmental Sciences, National Chung Cheng University, Chiayi, Taiwan; 8 Division of Infectious Diseases, Department of Internal Medicine, Kaohsiung Veterans General Hospital, Kaohsiung, Taiwan; 9 School of Medicine, College of Medicine, National Sun Yat-sen University, Kaohsiung, Taiwan; Institut de recherche pour le developpement, FRANCE

## Abstract

Melioidosis outbreaks in Taiwan frequently coincided with severe typhoons. Over a 20-year period, 782 cases of melioidosis were reported, with outbreaks often clustering in a specific hotspot area. We hypothesized that the unique hilly terrain in this hotspot trapped contaminated aerosols generated from northern to northwestern farming land within the area and restricted their spread beyond it. Across Taiwan, and particularly within the hotspot, weekly melioidosis incidence was significantly correlated with heavy rainfall and strong wind speed with time lags of 0, 1 and 2 weeks. When rainfall exceeded 200 mm and wind gust speeds reached over 20 m/sec, melioidosis outbreaks were frequently observed. Additionally, melioidosis incidence was associated with riverbank repair activities, indicating severe flooding caused by typhoons. Environmental confounding factors, such as CH₄, CO, NO, NO₂, NO_x_, O₃, particulate matter (PM)_10_, PM_2.5_, SO₂, and total hydrocarbon (THC), fluctuated seasonally but were not correlated with melioidosis cases. Aerosol sampling revealed that concentrations of contaminated aerosols were markedly higher north of the hill, where farming land was more prevalent, compared to the south, which had no farming land and was primarily residential. In addition to heavy rainfall and strong wind speed, shifts in wind direction from southwesterly to northwesterly during typhoons appeared to concentrate aerosols in the northern area but not in the south. Higher seropositive rates for *Burkholderia pseudomallei* antibodies in northern residents, compared to those in the south, further suggested increased exposure to pathogen-laden aerosols in the northern hotspot. This study demonstrated that heavy rainfall, combined with strong directional winds, generated high concentrations of contaminated aerosols from farming land in specific hilly terrains, leading to localized melioidosis outbreaks. It provided a valuable example of geographical, and climatic factors driving the formation of melioidosis hotspots in subtropical regions such as southern Taiwan.

## Author summary

Melioidosis, a disease caused by the environmental bacterium *Burkholderia pseudomallei*, represented a significant health risk in tropical and subtropical regions due to its high mortality rate and sudden outbreaks. This study explored the hypothesis that climate and geography affected the spread of melioidosis in southern Taiwan. We found that specific environmental conditions, such as heavy rainfall and strong winds in certain hilly areas, increased the risk of exposure to airborne *B. pseudomallei*. Higher infection rates were linked to riverbank repairs following severe typhoon-induced flooding. Outbreaks often occurred when rainfall exceeded 200 mm and wind gust speeds surpassed 20 m/sec, conditions commonly associated with typhoons. Wind direction also influenced bacterial spread, as seasonal southwesterly winds shifted to typhoon-driven northwesterly winds, but the hills served as a barrier, limiting the spread of aerosolized bacteria beyond these regions. Seropositivity to *B. pseudomallei* was higher in populations north of the hills than in those to the south. These findings highlighted the importance of environmental monitoring in controlling infectious diseases affected by climate and geography, especially in areas prone to extreme weather.

## Introduction

Melioidosis, an emerging and neglected infectious disease caused by *Burkholderia pseudomallei*, is endemic to tropical and subtropical regions worldwide [1–2]. Humans primarily contract *B. pseudomallei* through subcutaneous exposure, inhalation, or ingestion of contaminated soil or water [3]. In endemic regions, melioidosis cases often clustered following extreme weather events such as heavy rainfall, cyclones, typhoons, or tsunamis [4–6]. These events cause severe flooding, which brings *B. pseudomallei* to the surface from deeper soil layers, significantly increasing the risk of human or animal exposure [5,7]. Human melioidosis cases and the environmental isolation rates for *B. pseudomallei* rose significantly after flooding or extreme weather events, with genetic analysis often confirming identical strains in both environmental and human samples [8–10].

Human-to-human transmission of melioidosis is rare; however, pulmonary melioidosis can be induced through aerosolization of fine particles contaminated with *B. pseudomallei* in animal models [11–13]. Although *B. pseudomallei* was commonly isolated from soil and water in endemic regions, detecting contaminated aerosols remained challenging [14]. The pathogen may exist transiently in the air, as it was sensitive to sunlight and dryness, and aerosolization occur only under specific conditions, such as strong perturbations that bring *B. pseudomallei* to the surface from deep soil layers. Notably, aerosolized *B. pseudomallei* were documented after typhoons in Taiwan and Hong Kong, suggesting that extreme weather facilitated airborne transmission [9–10].

*B. pseudomallei* was isolated from soils and waters in farming areas in the endemic areas, with isolation rates usually peaking after typhoons and flooding events [15–18]. Since 2003, Taiwan has experienced repeated melioidosis outbreaks in a specific hotspot following severe typhoons [19–20]. The geography of this hotspot included a unique hill formation that likely disrupted wind patterns, particularly northwesterly winds, creating zones of higher melioidosis incidences in the north and lower incidences in the south. We hypothesized that the hills act as a barrier, concentrating contaminated aerosols within the northern endemic area and limiting transmission to the adjacent southern non-endemic region. This study presents a model illustrating that specific terrain features, when combined with heavy rainfall, strong winds, and favorable wind direction, contribute to the formation of melioidosis hotspots. These findings further emphasize their potential alignment with the Sustainable Development Goals (SDGs), particularly SDG 3 (Good Health and Well-being) and SDG 13 (Climate Action), by highlighting the connection between environmental factors, public health outcomes, and climate resilience.

## Materials and methods

### Ethics statement

Patient data were anonymized in compliance with Taiwan’s Personal Information Protection Act, and the study received approval from the Kaohsiung Veterans General Hospital IRB (AF01–007/108.3).

### Data collection

Weekly melioidosis case data from January, 2003 to October, 2024 were sourced from the Taiwan CDC. In parallel, the climatic data, including rainfall, wind speeds, and wind direction, were obtained from Taiwan Central Weather Bureau. Environmental pollutant data (CH₄, ppm; CO, ppm; NO, ppb; NO₂, ppb; NO_x_, ppb; O₃, ppb; particulate matter [PM_10_, µg/m³; PM_2.5_, µg/m³]; SO₂, ppb; and THC [total hydrocarbon, ppm]) were collected from the open databases of Ministry of Environment (Taiwan). Data on the total lengths of seawall or riverbank repairs from 2008 to 2023 were obtained from reports by the Water Resource Agency, Taiwan.

### Aerosol sampling and analysis

Aerosols were collected using sterile Teflon membranes and an air pump operating at 20 L/min for 2 hours, either at 1–2 day intervals or daily during changes in climatic conditions, particularly during typhoon events [20]. The membranes were immersed in 1 mL of phosphate-buffered saline (PBS), flushed with an additional 1 mL of PBS, and subjected to sonication. The solution was then centrifuged at 4,500 × g for 10 minutes, and total DNA was extracted from the sediment using the QIAamp DNA Mini Kit (QIAGEN, GmbH, Hilden, Germany). *B. pseudomallei*-specific DNA was targeted by amplifying a 115-bp region within *orf2* of the type III secretion system gene [20].

Quantitative PCR (qPCR) was conducted on a 7900 HT Fast Real-Time PCR System (Applied Biosystems Inc., Foster City, CA, USA). Positive control solutions containing approximately 10² CFU/mL of *B. pseudomalle*i (verified by plating and serial dilution) and sterile water as a negative control were included in parallel. The calibration curve, built with 10-fold serial dilutions of a standard DNA target (Mission Biotech Inc., Taipei, Taiwan), ranged from −3.6 to 3.1, achieving an efficiency of 90–110% and a linear regression coefficient of determination (r²) exceeding 0.996. The lower detection limit in this study was 6.94 copies/m³.

### Seroprevalence

Serum samples, distributed according to township, were collected weekly from anonymized leftover blood specimens of adults at the Out-Patient Department (OPD) at Kaohsiung Veterans General Hospital (KVGH), located near a hotspot in Taiwan, between 2019 and 2020. The study was reviewed and approved by the Institutional Review Board (IRB) of KVGH (Approval Number: AF01–007/108.3). As only anonymized leftover blood specimens were used, the IRB granted a waiver of informed consent. No personal identifiers were linked to the serum samples, ensuring participant anonymity. The study was conducted in accordance with the ethical principles outlined in the Declaration of Helsinki and adhered to IRB-approved protocols for data protection. At KVGH, an average of 21,000 people visited the OPD weekly, with approximately 5,000 undergoing blood examinations. Of these, around 2,000 leftover serum samples were utilized for the study. Based on a 3–5 fold prevalence of melioidosis over 15 years and population sizes, serum samples were randomly selected from leftover pools. In total, 1,526 serum samples were collected from the north of the hills and 2,062 from the south, achieving a statistical power of >0.99 (α = 0.05; calculated using the PWR package in R v4.4.1).

The presence of anti-*B. pseudomallei* flagella protein antibodies was determined using an indirect enzyme-linked immunosorbent assay (ELISA) as described by Chen et al. (2003) [21]. Ninety-six-well polystyrene microtiter plates were coated with flagellin (0.5 μg/mL, purified from *E. coli* DH5α harboring the recombinant plasmid pGEX4T-2/*fliC*) in a coating buffer (50 mM carbonate/bicarbonate, pH 9.6) and incubated overnight at 4°C. Afterward, the wells were blocked with 100 μL of 1 mg/mL bovine serum albumin (GIBCO, Grand Island, NY, USA) and washed using a saline-Tween solution (0.9% [wt/vol] NaCl and 0.05% [vol/vol] Tween 20 in PBS). Twofold serial dilutions of the serum samples were added to the wells and incubated at 37°C for 1 hour. Secondary antibodies, specifically anti-human immunoglobulin G conjugated with peroxidase (Zymed, South San Francisco, CA, USA), were used to detect bound antibodies. Color development was achieved using 100 μL of 1-Step Turbo tetramethylbenzidine ELISA substrate (Pierce Corp., Holmdel, NY, USA). The optical density at 450 nm (OD450) was measured using a microplate reader (Anthos 2020; Biocompare Inc., San Francisco, CA, USA). For this study, sera were considered positive for *B. pseudomallei* if, at a dilution of 1:256, the average OD450 of the sample exceeded the mean OD450 of the negative controls by at least 2 standard deviations, as determined.

### Statistical analysis

In the preliminary analysis, multiple linear, Poisson and negative binomial regression models were used to evaluate the correlation between disease incidence or aerosol concentration and meteorological factors, including rainfall, wind speed, and their interaction. All models yielded consistent results, showing significant differences. Considering the case distribution, high zero counts in the data, and Akaike Information Criterion (AIC) values, a zero-inflated Poisson regression model was ultimately determined to better fit the hypothesis and was therefore applied in this study (SPSS v29.0.2.0). Simple linear regression was conducted to analysis the correlation between melioidosis cases and rainfall exceeded 200 mm, or wind gusts surpassed 20 m/sec during typhoon events, as well as between melioidosis cases and the length of riverbank repairs. The Incidence Rate Ratio (IRR) with 95% Confidence Interval (CI) from zero-inflated Poisson regression, and R² and β values (α = 0.05) from simple linear regression, were reported.

Rainfall was described as the total precipitation during the specified time period. Wind speeds were analyzed using different metrics, including the average wind speed, maximum wind speed during the specified period, and wind gust speeds during typhoons for interpretative purposes. The proportion (%) of wind directions was visualized using a wind rose plot. Group differences were assessed using either the Mann-Whitney U test or Student’s t-test, depending on the data distribution.

## Results

### Melioidosis during typhoon events

**Fig 1:**
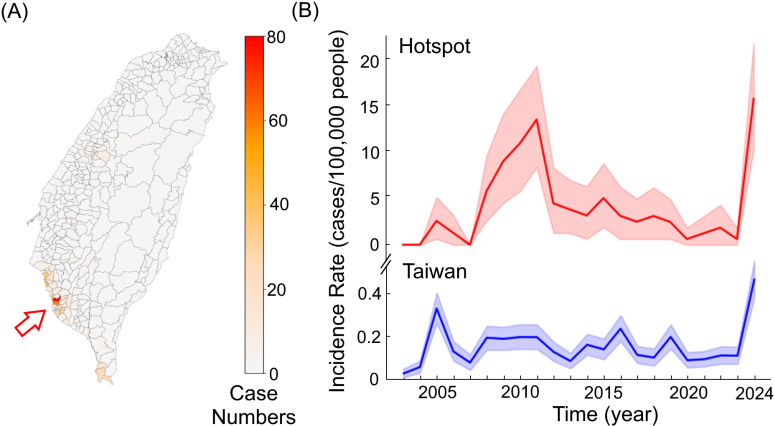
Distribution of melioidosis. The geographical distribution of melioidosis cases (n=782) across townships in Taiwan is illustrated, with colors ranging from light to dark indicating case numbers from low to high. The arrow highlights the hotspot of melioidosis in southern Taiwan (A). The annual incidence rates in the hotspot (red) and Taiwan (blue) are depicted, with shaded areas representing 95% CI. The basemap was derived from open-access shapefiles provided by the Taiwan Government Open Data Platform (https://data.gov.tw, Open Government Data License, version 1.0).

Since 2003, a total of 782 melioidosis cases were reported by hospitals in Taiwan over a 20-year period. Of these, 144 cases were clustered within a hotspot region (Fig 1A). The annual incidence rate across Taiwan ranged from 0.02 to 0.47 cases per 100,000 people; however, this rate spiked to 22.04 cases per 100,000 people in the identified hotspot (Fig 1B).

**Fig 2:**
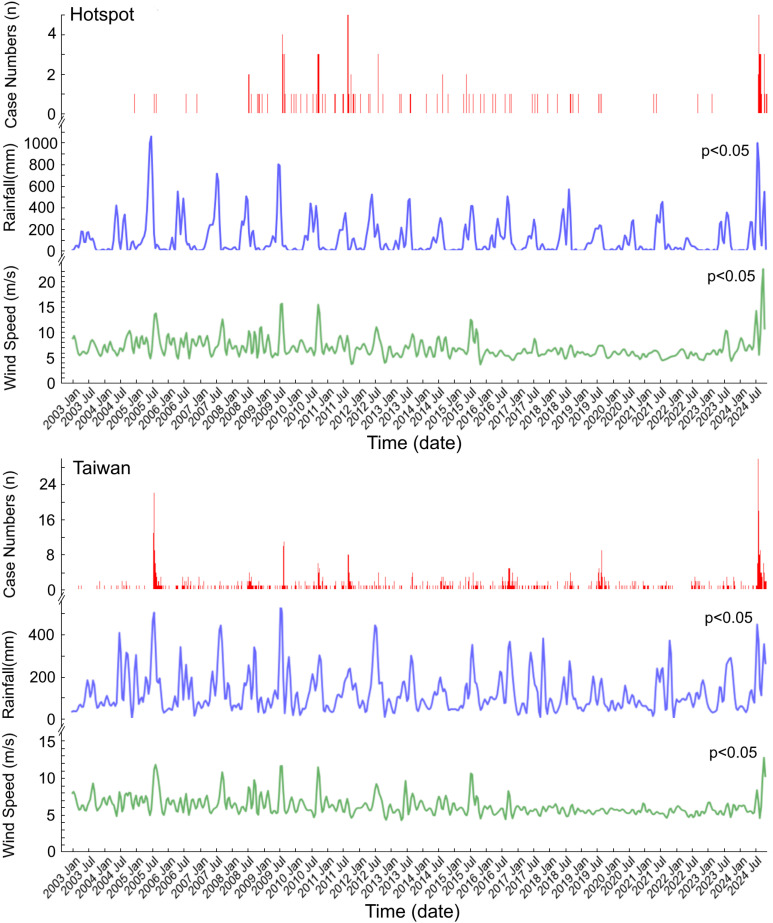
Melioidosis cases, rainfall, and wind speed from January 2003 to October 2024. Weekly melioidosis cases (n, red), rainfall (mm, blue), and maximum wind speeds (m/sec, green) are presented for the hotspot (upper panel, n=144) and Taiwan (lower panel, n=782). Significant correlations (p < 0.05) with time lags of 0, 1 and 2 weeks were identified using a zero-inflated Poisson regression model (IRR, wind speed, 1.14 [95% CI: 1.07–1.22] in Taiwan, 1.10 [95% CI: 1.03–1.18] in hotspot, per 1 m/sec; IRR, rainfall, 1.15 [95% CI: 1.10–1.21] in Taiwan, 1.12 [95% CI: 1.09–1.14] in hotspot, per 50 mm).

**Fig 3:**
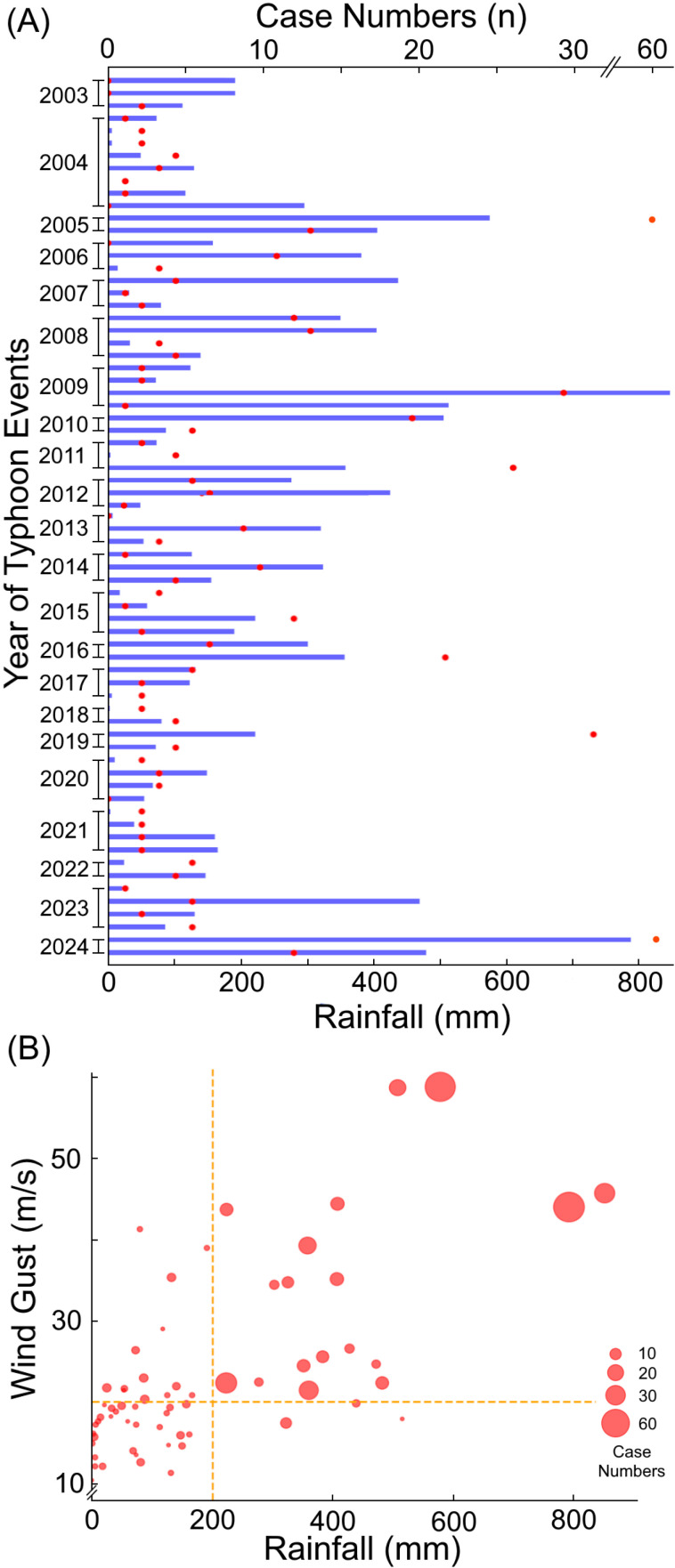
Melioidosis case numbers, rainfall, and wind gusts during typhoon events. From January 2003 to October 2024, a total of 70 invasive typhoons making landfall in Taiwan and 462 melioidosis cases during these typhoon events are shown in chronological order (left). The number of melioidosis cases (n, red dots) and rainfall (mm, blue line) occurred in each typhoon period are illustrated (A). Panel (B) shows the distribution of melioidosis cases (red dots) in relation to rainfall (mm) and wind gusts (m/sec), with red dots representing individual typhoon periods. The sizes of the red dots correspond to case numbers, ranging from 1 (smallest) to 62 (largest). A total of 6 dots are not visible in the figure because no cases occurred in these 6 typhoons (B).

Melioidosis cases exhibited a sudden increase following typhoon invasions. Using a zero-inflated Poisson regression model, we found that both rainfall and maximum wind speed were significantly correlated with weekly melioidosis incidence rates in both Taiwan and the hotspot over the 20-year period (Fig 2, p < 0.05; with time lags of 0, 1, or 2 weeks; IRR, wind speed, 1.14 [95% CI: 1.07–1.22] in Taiwan, 1.10 [95% CI: 1.03–1.18] in hotspot, per 1 m/sec; IRR, rainfall, 1.15 [95% CI: 1.10–1.21] in Taiwan, 1.12 [95% CI: 1.09–1.14] in hotspot, per 50 mm). The primary rainy seasons of Taiwan included the plum rains in the spring and typhoons in the summer, though these seasons sometimes overlapped. Since 2003, the Taiwan Central Weather Bureau has issued sea warnings for 117 typhoons, but not all made landfall, with only 70 making landfall in Taiwan. A total of 462 cases occurred during these typhoon events (Fig 3A). When rainfall exceeded 200 mm and wind gusts surpassed 20 m/sec (a typhoon is defined as having wind gusts of 17.2 m/sec or more), a noticeable increase in melioidosis cases was observed (Fig 3B). Linear regression analysis revealed that melioidosis cases were respectively correlated with rainfall and wind speed (cases vs. rainfall: r² = 0.51, β = 0.71, p < 0.05; cases vs. wind speed: r² = 0.35, β = 0.59, p < 0.05).

### Melioidosis and river flooding

**Fig 4:**
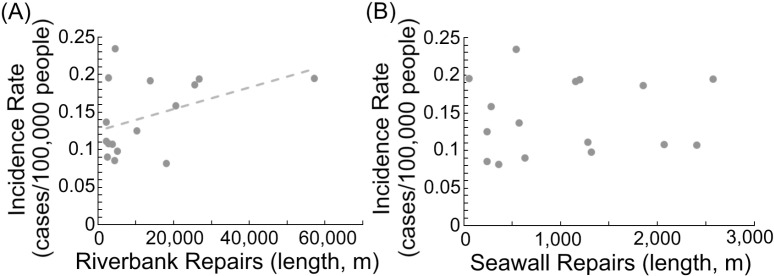
Association between melioidosis incidence and bank repairs. The association between melioidosis incidence and the lengths of riverbank repairs (m) (r2 = 0.18, β = 0.43, p < 0.05) (A) and seawall repairs (m) (B) is illustrated based on official reports from 2008 to 2023. The dotted line represents the regression slope.

Flooding in Taiwan typically resulted from seawater backflow or river overflow. Annual repairs to seawalls and riverbanks reflected the extent of damage caused by typhoon events. Based on official reports of repairs from 2008 to 2023, we found that the total lengths of riverbank repairs were associated with melioidosis incidence (Fig 4A; r^2^ = 0.18, β = 0.43, p < 0.05). However, no such correlation was observed for seawall repairs (Fig 4B). River flooding was suggested to play a role in the spread of environmental *B. pseudomallei*, thereby increasing the risk of melioidosis transmission.

### Aerosol transmission, hill terrain, and climatic factors

**Fig 5:**
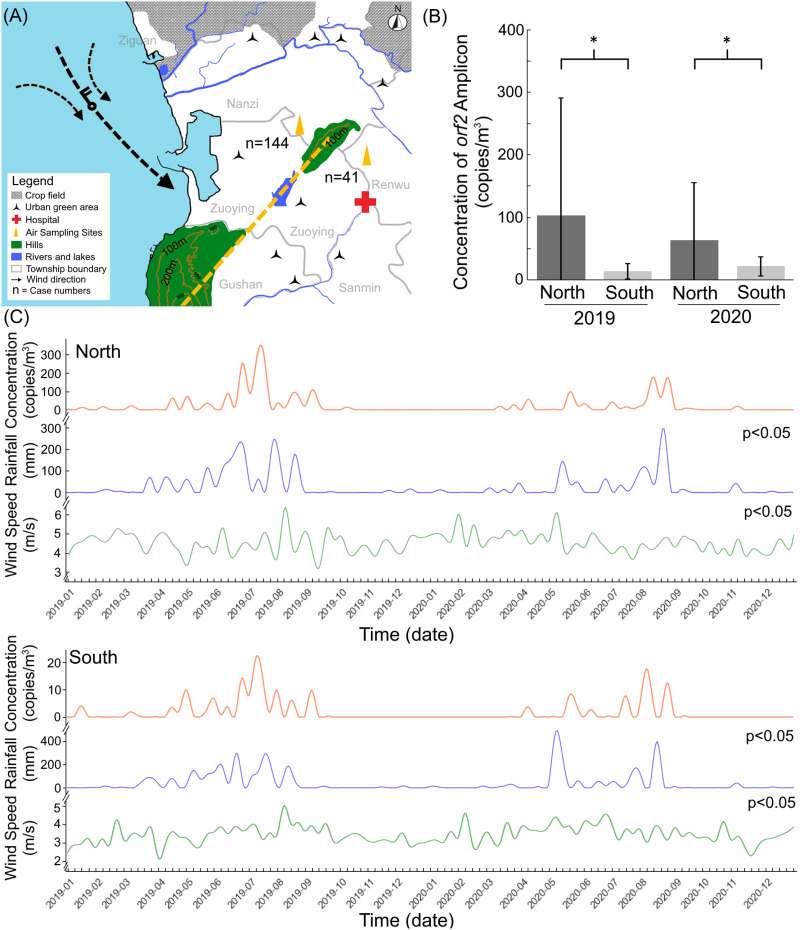
Hypothesis and distribution of specific *orf2* amplicons, rainfall, and wind speed in the north and south of the hills. It is hypothesized that *B. pseudomallei*-contaminated aerosols were generated from farming soils in the north to northwestern region and transported southward to the hotspot during typhoon events, with the hills (green) acting as a barrier that interrupted further dissemination. Panel (A) illustrates the wind direction (black wind barbs indicated) during the typhoon period and the melioidosis cases (n) occurring in the north and south of the hills. The dotted line indicates the highest points of the two hills. The triangles (yellow) indicate the air sampling sites. Panel (B) shows the average detectable concentrations of *B. pseudomallei*-specific *orf2* amplicons (copies/m^3^) in the north and south of the hills from 2019 to 2020. An asterisk (*) indicates statistical significance (p < 0.05). Panel (C) presents the daily average concentrations of the specific DNA (copies/m^3^), rainfall (mm), and wind speed (m/sec) are shown (C), with significance correlations (p < 0.05) observed for time lags of 3 and 4 days (IRR, wind speed, 1.04 [95% CI: 1.02–1.06] in north sides, 1.20 [95% CI: 1.11–1.29] in south sides, per 1 m/sec; IRR, rainfall, 1.45 [95% CI: 1.44–1.46] in north sides, 1.08 [95% CI: 0.99–1.19] in south sides, per 10 mm). The basemap was derived from open-access shapefiles provided by the Taiwan Government Open Data Platform (https://data.gov.tw, Open Government Data License, version 1.0). Crop fields and urban green areas were manually labeled based on reference locations identified from government datasets.

A question arose as to why typhoons, which affected areas with radii exceeding 100 Km and caused widespread disasters, resulted in melioidosis cases being clustered within a small 7.5 km² hotspot [10]. Over 20 years, 144 melioidosis cases were recorded in this hotspot, located between farming fields to the north or northwestern and residential areas near the hills to the southeastern. In contrast, areas outside the hotspot, southward beyond the hills, reported only 41 cases over the same period. It was hypothesized that *B. pseudomallei*-contaminated aerosols were generated from farming soils in the north to northwestern region and transported southward to the hotspot by heavy rain and strong winds, with the hills acting as a barrier to interrupted further dissemination (Fig 5A).

To test this hypothesis, aerosols were collected from both the northern and southern sides of the hill, and the concentration of *B. pseudomallei*-specific *orf2* amplicons was measured between 2019 and 2020. The average detectable concentration of these amplicons was significantly higher in the north compared to the south (Fig 5B, p < 0.05). Furthermore, the presence of daily contaminated aerosols strongly correlated with heavy rain and maximum wind speed on both the north and south sides (Fig 5C, p < 0.05; with time lags of 3 and 4 days; IRR, wind speed, 1.04 [95% CI: 1.02–1.06] in north sides, 1.20 [95% CI: 1.11–1.29] in south sides, per 1 m/sec; IRR, rainfall, 1.45 [95% CI: 1.44–1.46] in north sides, 1.08 [95% CI: 0.99–1.19] in south sides, per 10 mm).

**Fig 6:**
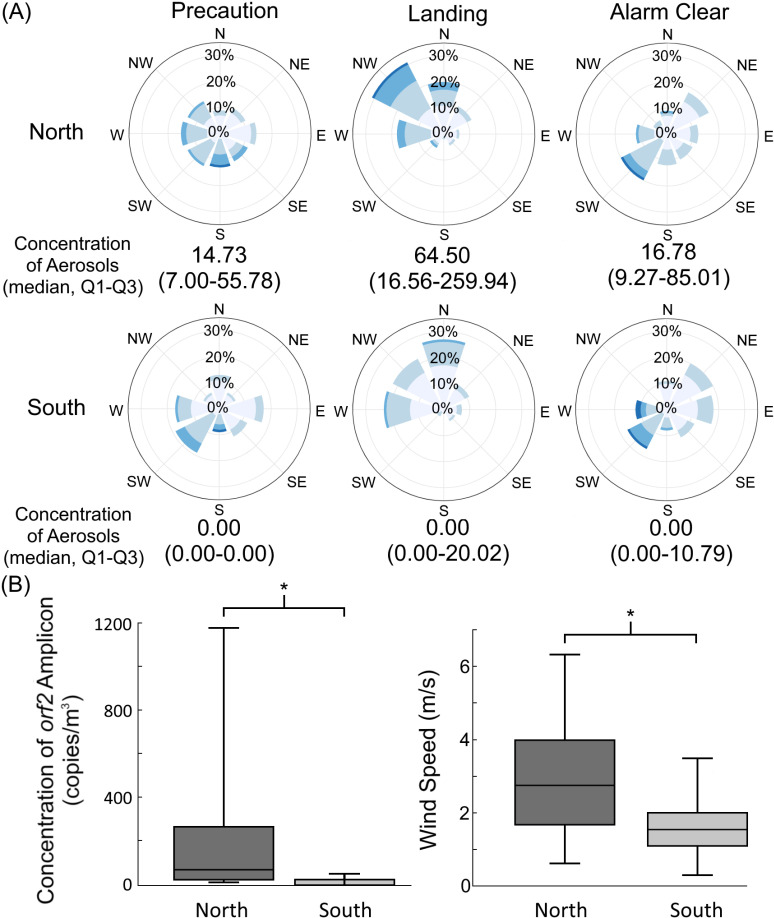
Changes of wind direction, wind speed and specific *orf2* amplicons during typhoon events for a range of years (2019–2020, n **=6).** The figure illustrated the proportion (%) of wind directions, average wind speed (m/sec), and the concentration of contaminated aerosols (median, Q1–Q3; copies/m³) during three phases: precaution, typhoon landing, and three days after the alarm was cleared. Shades of blue, from dark (6-8 m/sec, largest) to light (0-2 m/sec, smallest), divided into 4 intervals, represent decreasing average wind speeds per hour (A). Comparison of aerosol concentrations (median values) between the north and south sides of the hills is illustrated (B, left), and the comparison of average northwesterly wind speeds per hours (median values) between the same regions, during typhoon landing, is illustrated (B, right). An asterisk (*) indicates statistical significance (p < 0.05).

Typically, southwesterly winds dominated the rainy summer season, while northeasterly winds prevailed during the dry winter season. Although *B. pseudomallei* favorably grew in the moisture north to northwestern farming land; however, during typhoons, wind direction shifted to a northwesterly flow due to the cyclone’s counterclockwise rotation in southern Taiwan. Observations from typhoon events from 2019 to 2020, the hilly terrain served as a natural barrier, significantly reducing the proportion of northwesterly winds and average wind speeds in the south compared to the north. The presence of northwesterly winds was associated with increased concentrations of contaminated aerosols (Fig 6A). During typhoons, the hilly terrain effectively reduced both aerosol contamination levels and wind speeds with median values being lower in the south compared to the north (Fig 6B).

Several confounding factors, including CH₄, CO, NO, NO₂, NO_x_, O₃, PM_10_, PM_2.5_, SO₂, and THC, fluctuated seasonally and were evaluated for their correlation with melioidosis cases (S1 Fig). However, in 2012, 2014, and 2017, when melioidosis incidence exceeded 2.51 cases per 100,000 population, no significant correlation was found between these factors and case occurrences. Conversely, in 2003–2004 and 2007, these factors increased during seasonal changes, although no melioidosis cases were reported in hotspot (S1 Table).

### Seroprevalence in hotspot

To investigate the increased exposure to *B. pseudomallei* in the hotspot area with high concentrations of *B. pseudomallei* amplicons, a total of 1,526 and 2,062 serum samples were collected from residents in the north and south of the hill, respectively, between 2019 and 2020. Approximately 40.1% (612/1,526) of the serum samples from the northern area tested positive for *B. pseudomallei* flagella protein, compared to 8.5% (176/2,062) positivity in the southern area (Table 1). These findings suggested that people in the north, who were directly exposed to contaminated aerosols, faced a significantly higher risk of *B. pseudomallei* exposure than those living south of the hill, where the terrain acted as a barrier to northerly wind-driven aerosol transmission.

**Table 1:** Seropositivity to melioidosis in the northern and southern regions of the hills.

	Distribution	
	North of hill	South of hill
Total population	416,675	569,988
Sampling size	1,526	2,062
Sampling ratio (%)	0.37	0.36
Positive samples	612	176
Positivity (%)	40.1	8.5

## Discussion

Heavy rainfall and flooding events have been associated with clusters of melioidosis cases. For example, sudden increases in melioidosis cases were observed following several major weather events, such as a typhoon in Taiwan [7,22], a tsunami in Thailand [23], a tropical cyclone in Northern Australia [24], and intense rainfall in Singapore and Northern Hainan, China [26–27]. Climate change has been thought to contribute to the growth of *B. pseudomallei* in the environment, and floodwaters have increased the spread of melioidosis into new areas [28–29]. In addition to rainfall, other climatic factors such as humidity, dew point, and cloud cover have been statistically correlated with melioidosis incidence [24,25,29]. These factors increased soil moisture, which supported the growth of *B. pseudomallei* [5].

In this study, we focused on a hotspot in Taiwan, where rainfall, combined with wind speed, particularly during typhoon events, showed a significant correlation with melioidosis incidence over a 20-year period. Case clusters of melioidosis in Taiwan and Hong Kong were reported to result from airborne transmission [10–11]. Wind, acting as a vector for *B. pseudomallei*, facilitated the dissemination of melioidosis, especially under favorable wind directions. We found that shifts in wind direction from the usual seasonal southwesterly to typhoon-driven northwesterly winds were associated with increased melioidosis cases in the region.

Several confounding factors, including CH₄, CO, NO, NO₂, NO_x_, O₃, PM_10_, PM_2.5_, SO₂, and THC, were not found to be directly correlated with the annual incidence of melioidosis. However, it is noteworthy that human-made air pollution has been linked to the exacerbation of bacterial respiratory diseases, and climate change can further aggravate air pollution [30–31].

Evidence of identical *B. pseudomallei* sequence types (ST) in both aerosol and human samples suggested that airborne transmission occurred in Taiwan’s melioidosis hotspots [10]. Studies comparing flat and hilly regions in Taiwan have shown varying melioidosis case patterns correlated with rainfall, wind speed, and direction [22]. In this study, hill terrain likely played a dual role of restricting aerosol transmission to the south and accumulating aerosols in the north of the hills, further influencing the distribution of *B. pseudomallei*-contaminated aerosols, across northern hotspot and southern non-hotspot areas. In case of restricting aerosol transmission, the hills acted as a natural barrier to northwesterly winds, limiting the southward spread of aerosols and resulting in a lower concentration of *B. pseudomallei*-contaminated aerosols in the southern non-hotspot areas. People dwelling the south reduced the chances of exposure to *B. pseudomallei* so that the seropositive rate were low. In term of accumulating aerosols, the hotspot faced north to northwest, adjacent to agricultural lands with scattered feedlots for cattle, sheep, and swine amid large crop fields. This layout, combined with heavy rain and strong northwesterly winds during typhoon seasons, likely generated aerosols contaminated with *B. pseudomallei* from northern or northwestern soil or water, which then enveloped the hotspot. Since the hill interrupted aerosol transmission to the south, it caused aerosols to accumulate on the northern side, where wind patterns and topography trapped them. This led to higher exposure and increased seropositivity among people in these hotspot areas. Actually, we found that the concentration of aerosols accumulates over time and typically peaks within approximately 3–4 days during a typhoon event.

Severe weather events often lead to flooding, which has been associated with human and animal cases of melioidosis in several regions [5,15,32]. Rivers, which may serve as reservoirs, are thought to play a role in the distribution and waterborne spread of *B. pseudomallei* [15,33]. Sites with high rates of *B. pseudomallei* have been found near large wetlands connected to rivers [34]. Genetic lineages have demonstrated that the geographical spread of *B. pseudomallei* is influenced by terrain slope, altitude, and river flow [35]. Our findings also suggested that riverbank repairs following typhoon-induced flooding were linked to increased melioidosis incidence, emphasizing the importance of flood control in preventing melioidosis outbreaks during typhoon events. Despite airborne transmission being highlighted in this study, people residing south of the hill, or in some cases to the north, likely acquired melioidosis through direct contact with *B. pseudomallei* in the soil or water while traveling, working or during flooding event. Indeed, low isolation rates of *B. pseudomallei* from environment have been previously reported south of the hills [10].

In summary, this study highlighted that melioidosis cases in a hotspot with unique hilly terrain were strongly associated with typhoon conditions, where heavy rainfall combined with strong winds from specific directions resulted in high concentrations of *B. pseudomallei*-contaminated aerosols. This environment increased exposure risks for local residents. The hotspot serves as a representative case for understanding melioidosis risk in subtropical (and tropical) regions, like Taiwan, and offers insight into the geographic and climatic factors contributing to the formation of melioidosis hotspots.

## Supporting information

S1 Fig Seasonal change of confounding factors in this study. (TIF)

S1 Table Association between incidence rates of melioidosis and environmental confounding factors. (PDF)
