# Supplementary figures and images for: Geographical and climatic contributions to melioidosis hotspot formation in Southern Taiwan
Source: PLoS Negl Trop Dis. 2025 Apr 10;19(4):e0012958. doi: 10.1371/journal.pntd.0012958 (PMC12080920; doi:10.1371/journal.pntd.0012958)

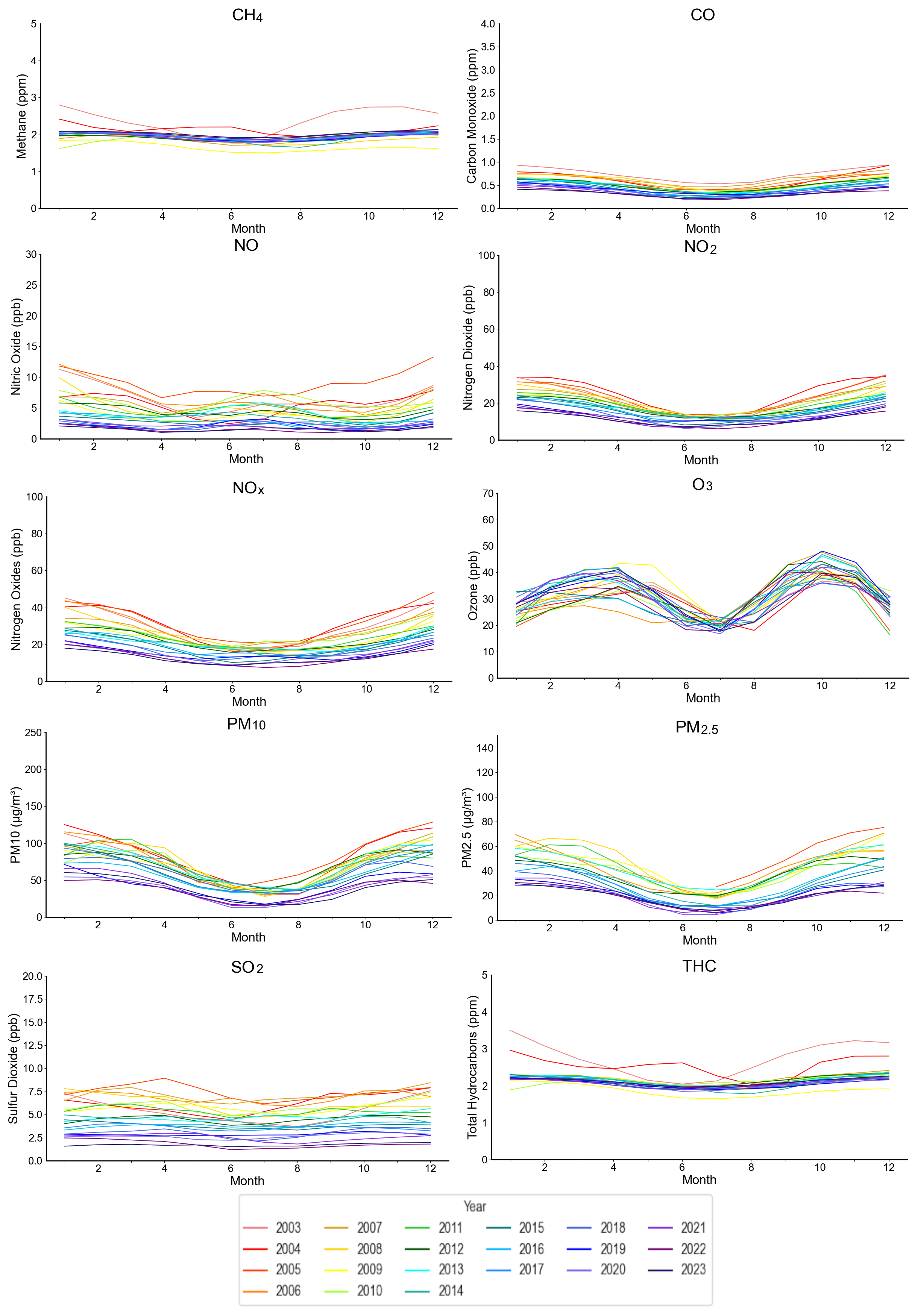

Supplement: S1 Fig — (TIF) [file pntd.0012958.s001.tif]
