# Supplementary material for: Geographical and climatic contributions to melioidosis hotspot formation in Southern Taiwan
Source: PLoS Negl Trop Dis. 2025 Apr 10;19(4):e0012958. doi: 10.1371/journal.pntd.0012958 (PMC12080920; doi:10.1371/journal.pntd.0012958)
Supplement: S1 Table — (PDF) [file pntd.0012958.s002.pdf]

S1 Table. Association between Incidence Rates of Melioidosis and Environmental Confounding Factors.

	Incidence Rate (95% CI)	CH_4	CO	NO	NO_2	NO_x	O_3	PM_{10}	$PM_{2.5}$	SO_2	THC
Whole Period*											
2003-2023		Negative	Negative		Negative	Negative		Negative	Negative		Negative
Each Year**											
2003	0.00 (0.00-0.00)										
2004	0.00 (0.00-0.00)										
2005	2.60 (0.65-5.20)		Negative	Negative	Negative	Negative		Negative	Negative	Negative	
2006	1.30 (0.00-3.25)										
2007	0.00 (0.00-0.00)										
2008	5.85 (2.60-9.76)	Negative				Negative				Negative	Negative
2009	9.11 (4.55-14.31)	Negative	Negative		Negative		Negative	Negative	Negative		Negative
2010	11.06 (5.85-16.91)	Positive					Positive				Positive
2011	13.66 (8.46-19.51)			Negative	Negative	Negative	Positive			Negative	
2012	4.52 (1.29-8.39)										
2013	3.84 (1.28-7.05)			Negative			Positive				
2014	3.19 (0.64-6.37)										
2015	5.08 (1.91-8.89)	Negative	Negative		Negative	Negative		Negative	Negative		Negative
2016	3.16 (0.63-6.32)			Negative							
2017	2.51 (0.63-5.03)										
2018	3.13 (0.63-6.26)			Positive			Negative		Negative		
2019	2.49 (0.62-4.99)	Negative	Negative		Negative	Negative		Negative	Negative		Negative
2020	0.62 (0.00-1.86)										
2021	1.24 (0.00-3.09)										
2022	1.86 (0.00-4.34)	Negative	Negative	Negative	Negative	Negative			Negative		Negative
2023	0.62 (0.00-1.85)										

*: Significance $p<0.05$; negative correlation between incidence rate and individual confounding factor across studied years.

**: Significance $p<0.05$; negative or positive correlation between incidence rate and individual confounding factor in the indicated year.
